# Straining Flow Spinning of Artificial Silk Fibers: A Review

**DOI:** 10.3390/biomimetics3040029

**Published:** 2018-10-05

**Authors:** José Pérez-Rigueiro, Rodrigo Madurga, Alfonso M. Gañán-Calvo, Gustavo R. Plaza, Manuel Elices, Patricia A. López, Rafael Daza, Daniel González-Nieto, Gustavo V. Guinea

**Affiliations:** 1 Centro de Tecnología Biomédica, Universidad Politécnica de Madrid, 28223 Pozuelo de Alarcón, Madrid, Spain; rmadurga@gmail.com (R.M.); gustavo.plaza@upm.es (G.R.P.); m.elices@upm.es (M.E.); patricia.lopez@ctb.upm.es (P.A.L.); rafael.daza@upm.es (R.D.); daniel.gonzalez@ctb.upm.es (D.G.-N.); 2 Departamento de Ciencia de Materiales, ETSI Caminos, Canales y Puertos, Universidad Politécnica de Madrid, 28040 Madrid, Spain; 3 Biomedical Research Networking Center in Bioengineering, Biomaterials and Nanomedicine (CIBER-BBN), Madrid, Spain; 4 Departamento de Ingeniería Aeroespacial y Mecánica de Fluidos, Escuela Técnica Superior de Ingenieros, Universidad de Sevilla, 41092 Sevilla, Spain; amgc@us.es; 5 Departamento de Tecnología Fotónica y Bioingeniería, ETSI Telecomunicaciones, Universidad Politécnica de Madrid, 28040 Madrid, Spain

**Keywords:** silk, regenerated fibers, fibroin, spidroin

## Abstract

This work summarizes the main principles and some of the most significant results of straining flow spinning (SFS), a technology developed originally by the authors of this work. The principles on which the technology is based, inspired by the natural spinning system of silkworms and spiders, are presented, as well as some of the main achievements of the technique. Among these achievements, spinning under environmentally friendly conditions, obtaining high-performance fibers, and imparting the fibers with emerging properties such as supercontraction are discussed. Consequently, SFS appears as an efficient process that may represent one of the first realizations of a biomimetic technology with a significant impact at the production level.

## 1. Introduction

Like any great story, the study of spider and silkworm silks can be contemplated from a number of different perspectives [1], all of them fascinating. From its first appearance in the evolution of early Araneae [2] and the independent evolutionary event [3] that led to the production of silk fibers by some insects, most conspicuously from the Lepidoptera order, to the production of one of the toughest materials [4], the study of silk-based materials offers a complex picture of lessons for very different scientific areas. The recognition of these lessons has converted the bioinspired production of artificial silk-like materials into one of the leading research lines in the field of biomimetics.

The main event in natural spinning, as observed in spiders and silkworms, is the transition from a protein solution to a high-performance solid fiber. This process occurs in fractions of second and proceeds under extremely biosustainable, and thus environmentally friendly, conditions [5]. The success of this process depends on the careful design of the intervening elements, as well as on their coordinated action. Thus, one of the key elements of spinning is the peculiar sequence of the silk proteins, known as fibroins (or as spidroins when referred specifically to spider silk proteins). All silk fibroins show a common organization characterized by a large number of repeats of shortmotifs: (GAGAGS)*_n_* in silkworm (*Bombyx mori*) silk [6], and A*_n_*, (GGX)*_n_* (where X is often tyrosine or glutamine), (GPG)*_n_* and (GA)*_n_* in spider silks [7]. In addition to these repetitive motifs, fibroins show two extremely conserved N- and C-terminal domains [8,9], that act as pH switches and control the conformation and assembly of the proteins [10]. In this regard, it is assumed that at high values of pH the conformation of these terminal domains tend to favor the soluble state of the proteins, possibly in a dimeric state, while a decrease in pH leads to the assembly of the proteins into larger structures [11].

The dependence of the fibroin conformation and assembly on pH is exploited during the spinning process by a careful control of the pH values [12,13], which vary from pH ≈ 8.0 in the gland sac, where the protein is stored, to pH ≈ 5.0 in the duct, where the fiber is formed. Additionally, it is also hypothesized that the concentration of certain ions such as PO_4_^3−^, Na^+^, and Cl^−^ [14,15] along the silk gland may also influence the assembly of the proteins. There is some debate on the exact nature of the fibroin assemblies in the gland and two possible models were proposed to account for the singular properties of the silk dope (fibroin solution), most significantly its low viscosity [16,17] in spite of the large protein concentration found in the gland. A first model assumes that the silk fibroins form anisotropic structures that behave as a lyotropic liquid crystal [18]. A second model proposes the organization of the fibroin proteins in micellar structures, that are deformed along the silk gland [19]. It is believed that the actual natural spinning process might share characteristics of both models [10].

A larger consensus is found on the last steps of the spinning process in which the mechanical stresses to which the proteins are subjected seem to play a critical role [20,21]. Thus, it is assumed that the proteins, previously assembled inside the silk gland, undergo relative displacements that induce the alignment of the (GAGAGS)*_n_* motif in silkworm silk and of the A*_n_* in spider silk, and lead to the formation of β-nanocrystals. The β-nanocrystals result from the piling up of β-pleated sheets [22,23,24] and impart the material its structural integrity [25].

The efficiency of the natural process, and the number of possible new applications that might benefit from the availability of bioinspired silk fibers, naturally arose the interest in the artificial production of these materials. In this regard, although a significant number of spinning processes were proposed and analyzed, all of them can be grouped in one of three possible strategies [26]: (1) Wet spinning (small variations of wet spinning are represented by dry spinning and dry jet/wet spinning), (2) electrospinning, and (3) biomimetic spinning. Initial attempts were based on the wet spinning process in which a fibroin solution interacts with a coagulating bath, so that the solvent molecules are removed, resulting in the assembly of the silk proteins and in the formation of the fiber. It is believed that the first description of a wet spinning process for silk dates back to 1960 [27], in which fibers were produced from an aqueous fibroin solution using ammonium sulphate as coagulant. Almost three decades later, a similar approach was used by Ishizaka et al. [28], using orthophosphoric acid (H_3_PO_4_) as solvent and a mixture of ammonium and sodium sulphate as coagulant. Subsequent attempts have varied the chemistry of the solvent or the coagulant while maintaining the principles of the process [29,30,31,32]. As indicated above, dry spinning represents a variation of wet spinning in which the solvent evaporates to a gaseous environment instead of being removed through the interaction between the dope and the coagulating bath. Artificial silk fibers with remarkable tensile properties were produced from highly concentrated fibroin solutions with this spinning technology [33,34]. Finally, in dry jet/wet spinning, the dry jet is forced to go through a small air gap before entering the coagulating bath. Such a dry jet/wet spinning procedure allowed the production of high-performance silk fibers from highly concentrated fibroin solutions containing *N*-methylmorpholine *N*-oxide (NMMO) to stabilize the dope [35].

An alternative to wet spinning and related processes is offered by electrospinning. In electrospinning, the dope jet is formed by the interaction of a dope solution with adequate electrical properties with an intense electric field, the solvent being evaporated into the environment [36]. Silk non-woven and mats can be efficiently produced through electrospinning, but the tensile properties of the individual fibers tend to be poor compared with those of the fibers produced through wet spinning and related techniques [37,38,39]. In addition to these conventional spinning techniques, the increased knowledge on the principles of the natural spinning process has led to the development of a number of biomimetic approaches that try to exploit these principles, for instance, the dependence of the natural assembly process on the pH of the solution [40,41,42,43,44]. 

In this context, straining flow spinning (SFS) was proposed and developed in a series of works [45,46,47,48,49] as a biomimetic approach to the production of artificial silk-based fibers. The SFS technique derives from flow focusing [50,51] and wet spinning technologies, and its main characteristic is that the physicochemical environment of the dope, as well as the mechanical stresses to which the fibroins are exposed, are controlled by the interaction of the dope with a jet of focusing fluid. The present review is an attempt at a quick introduction to the SFS technology, in which its biomimetic principles are highlighted. The details of the different aspects summarized here can be found in much greater detail in the references cited above. 

## 2. Materials and Methods in Straining Flow Spinning Studies

### 2.1. Preparation of the Dope

In our previous works on the development of the SFS technique, the dope consisted of a solution of silkworm (*Bombyx mori*) silk fibroin. Silk cocoons were degummed either in deionized water or in a Na_2_CO_3_ aqueous solution. Degumming in deionized water leads to fibroins with an average molecular weight of 100 kDa (high molecular weight regenerated fibroin), which decreases to 55 kDa (low molecular weight regenerated fibroin) in the Na_2_CO_3_ degumming solution. After degumming, the fibers were dissolved in a 9.4 M LiBr solution and dialyzed against deionized water. For a fibroin concentration higher than 4%, a reverse dialysis process against polyethyleneglycol (PEG) was used. Dopes with a fibroin concentration higher than 8% were prepared with 1 M CaCl_2_ to stabilize the solution.

### 2.2. Characterization of Silk Fibers

The optical micrographs of the fibers were obtained with a Leica DMI3000B microscope with a 40× objective. Scanning electron microscopy (SEM) micrographs were obtained in a field emission SEM Auriga Zeiss, coupled to an energy dispersive X-ray spectroscopy (EDS) X Flash Detector 5010 (Bruker, Billerica, MA, USA). The infrared spectra were obtained with a Nicolet iS5 spectrometer equipped with an iD5 ATR complement. Briefly, tensile tests were used for the mechanical characterization of the samples as described elsewhere [52]. Tensile tests were performed in an Instron 4411 tensile testing machine, and forces were measured with a precision balance (Precisa XT220A). Wet-stretched samples [53] were prepared from the as-spun fibers by stretching the fibers up to 90% of the breaking of the fiber in water, fixing the deformed length and allowing the fiber to dry before testing. If required, the full details of the Materials and Methods can be found in the original references [45,46,47,48,49]. 

## 3. Straining Flow Spinning: The Principles

**Figure 1:**
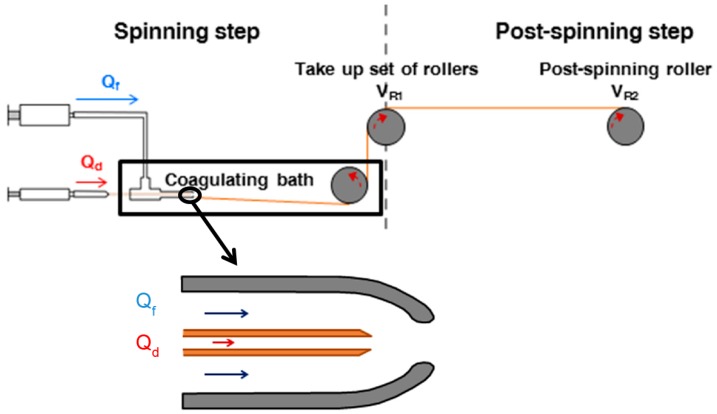
Schematic of a straining flow spinning process with its main elements. The inset shows a detail of the capillary–nozzle system in which the flow of the dope (*Q_d_*) and of the focusing fluid (*Q_f_*) are indicated. *V*_R1_: Velocity of the take-up roller; *V*_R2_: Velocity of the post-spinning roller.

The basic setup for a SFS process is schematically shown in Figure 1. The core of the technique consists of a coaxial capillary–nozzle system (see inset), so that the nozzle presents a convergent profile in the outlet region. The dope and the focusing fluid flow along the capillary and in the region confined by the outer wall of the capillary and the inner wall of the nozzle, respectively. Upon reaching the end of the capillary, a jet of dope is formed and surrounded by the focusing fluid. Finally, both the dope and the focusing fluid enter a coagulating bath, which may have the same or a different composition to that of the focusing fluid. The flow rates of the dope and of the focusing fluid are controlled by syringe pumps, and the fiber is retrieved from the coagulating bath and wound on a set of take-up rollers from where it can be retrieved. A post-spinning drawing step may be added to the process by including a post-spinning roller, to which the fiber is transferred from the take-up roller. Post-spinning drawing is the result of imposing a higher speed to the post-spinning roller than to the take-up roller, which leads to the stretching of the fiber during its processing.

The comparison of SFS with other spinning techniques reveals that one of the main singularities of the former is its versatility in terms of the number of parameters that control the spinning process. In this regard, as indicated in Table 1, the processing parameters in SFS can be divided into three groups: geometrical, hydrodynamic, and chemical. The geometrical parameters refer to the spatial assembly of the capillary–nozzle system and to the basic geometric features of their elements. The geometrical parameters comprise the inner and outer diameters of the capillary, the tapering angle at the end of the capillary, the distance between the end of the capillary and the nozzle outlet, the profile of the convergent region close to the nozzle outlet, and the nozzle outlet diameter. The hydrodynamic parameters correspond to the flow rates and speeds of the different components of the system and comprise flow rate of the dope, flow rate of the focusing fluid, velocity of the take-up roller, and in processes with a post-spinning drawing step, velocity of post-spinning drawing roller. The combined effect of the geometrical and hydrodynamic parameters controls the flow of both the dope and focusing fluids, and more importantly, their interaction. Finally, the chemical parameters comprise the composition of the dope, the composition of the focusing fluid and the composition of the coagulating bath. As indicated above, the focusing fluid and the coagulating bath often share a common composition, but SFS offers the possibility of using different compositions for both fluids.

## 4. Straining Flow Spinning as a Biomimetic Process

As indicated in the previous section, SFS is characterized by the large number of processing parameters that allow controlling the interaction between the dope and the focusing fluid, and eventually, the interaction between both fluids and the coagulating bath. It is precisely this interaction that lies at the basis of the development of SFS as a biomimetic technique. Conversely, the degrees of freedom present in SFS provide an optimum environment to discover what are the actual key factors that contribute to the extreme efficiency of the natural process.

**Figure 2:**
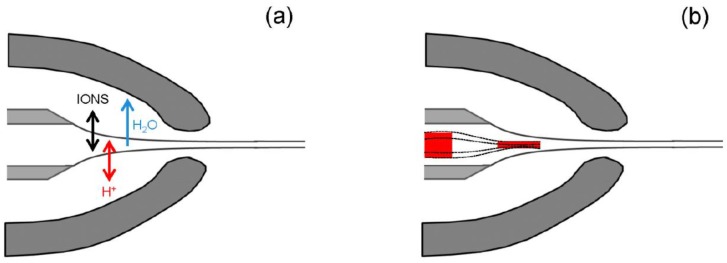
Schematic of the basic processes that result from the interaction between the dope jet and the focusing fluid. (**a**) Diffusion processes include the exchange of ions, including protons and the removal of water molecules from the dope to the focusing fluid. (**b**) The hydrodynamic interaction of both fluids results in the deformation of the dope jet.

Natural spinning in spiders and silkworms is the result of the combined effect of changes in the physicochemical environment and in the mechanical stresses to which the fibroin dope is exposed. Figure 2 schematically shows how the interaction between the dope and the focusing fluid mimics these changes in terms of the diffusion of chemical species between both fluids (Figure 2a) and of the deformations sustained by the dope jet (Figure 2b). As described for the natural spinning process, the transition from a protein solution to a solid fiber requires the removal of water molecules, initially present in the dope. This effect is favored by including dehydrating agents such as ethanol or PEG in the composition of the focusing fluid. In addition to the removal of water molecules, the formation of the solid fiber requires the concentration of several ions to vary, most importantly protons, along the silk gland and duct. This effect can be mimicked in the SFS process through the compositions of the dope and of the focusing fluid. Thus, the use of a mild acid in the composition of the focusing fluid is beneficial for the solidification of the fiber, probably through its effect on the pH switches of the fibroins. In turn, the removal of some components that stabilize the fibroin solution, such as CaCl_2_, may favor the formation of the solid fiber. In addition to the influence exerted by the chemistries of the different fluids, the flow lines and flow rates of the dope and of the focusing fluid also influence the diffusion by controlling, for instance, the rate to which fresh focusing fluid enters into contact with the dope jet. The influence of the flow lines and rates of the dope and focusing fluid is even more apparent on the deformation of the elementary volumes of the dope, which implies that these hydrodynamic features can be controlled through the geometrical and hydrodynamic parameters of the process. It should be mentioned, however, that the chemistry of the fluids also exerts a certain influence on the mechanical stresses exerted on the dope, in this case, mostly through the values of density and viscosity which, in turn, depend on the composition of the fluids.

The basic features indicated in Figure 2 will be presented in greater detail in the next section during the discussion of the influence of the different parameters on the outcome of the SFS process.

## 5. Influence of the Spinning Parameters on the Regenerated Silk Fibers

### 5.1. Composition of the Dope

Straining flow spinning allows the efficient production of fibers from aqueous solutions, so that water is the preferred solvent for the preparation of the fibroin dope. Besides, the full formulation of the dope is given by the sequence, or at least, by the average molecular weight of the fibroin proteins and by their concentration, as well as by the pH and by the possible addition of salts or other small chemical moieties to the solution.

When SFS is used to spin regenerated silkworm silk fibers, the average molecular weight of the fibroins depends on the particular conditions of the degumming process. Thus, larger proteins are obtained under milder degumming conditions so that, in particular, higher molecular weight proteins are retrieved if Na_2_CO_3_ is not added to the degumming solution. As a result of the damage induced in the proteins during degumming, the solubility of the silk proteins after degumming is smaller than that of the native protein. The decrease in solubility implies that regenerated silk solutions tend to be unstable at a protein concentration around 10% (*w*/*v*). Larger concentrations can be obtained, but a stabilizing agent such as NMMO [54,55] or CaCl_2_ [32,34] is required. If no buffer is added to the dope, the pH of the fibroin solution is in the range pH ≈5.0.

**Figure 3:**
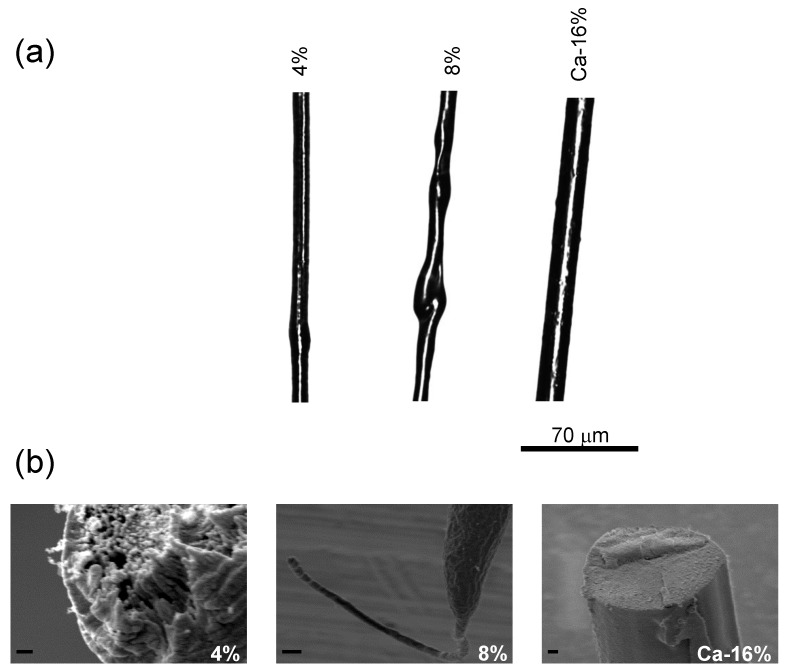
Morphology and fractographic analysis of regenerated silk fibers spun from dopes with different compositions. (**a**) Morphology of the fibers observed using optical microscopy. (**b**) Scanning electron microscopy (SEM) migrographs of the fracture surfaces of tensile tested samples. The percentage indicates the fibroin concentration in the dope and Ca indicates that the dope contains CaCl_2_ at 1 M concentration. The 4% sample shows the presence of voids in the fracture surface. The 8% sample shows the considerable necking undergone by the fiber prior to fracture. The 16% sample shows an essentially flat fracture surface. The scale bar in the SEM micrographs corresponds to 1 μm.

Figure 3 shows the influence exerted by the composition of the dope on the geometry of the fibers. Fibers produced from three different high molecular fibroin dopes (i.e., fibroin at a concentration of 4% (*w*/*v*) in water, fibroin at a concentration of 8% (*w*/*v*) in water, and Ca–fibroin at a concentration of 16% in a 1 M CaCl_2_ aqueous solution (Ca-16%)) are compared [49] in terms of their overall geometry and fracture surfaces after tensile testing. All fibers were produced using an 80% ethanol/20% 1 M acetic acid solution in water, so that the final acetic acid concentration was 0.2 M for both the focusing fluid and the coagulating bath. The rest of the spinning parameters were kept constant during the production of the different fibers.

The possibility of spinning from an aqueous dope with a low protein concentration such as 4% (*w*/*v*), one order of magnitude smaller than the concentration in the silkworm silk gland, is a feature characteristic of SFS. However, the fractographic analysis of the 4% sample, reveals the presence of some voids in the fracture surface (Figure 3b). Increasing the protein concentration to 8% leads to some irregularities in the fiber and a significant necking is observed when the fiber is tensile tested until breaking (Figure 3b). Finally, increasing the fibroin concentration up to 16% (*w*/*v*) was possible with the addition of 1 M CaCl_2_ to the dope. Samples produced from this dope are homogenous and their diameter is larger than that of the fibers spun from dopes with a smaller protein concentration (Figure 3a). The fractographic analysis of the Ca-16% sample reveals an essentially flat fracture surface (Figure 3b), where no void or clear defects can be identified.

**Figure 4:**
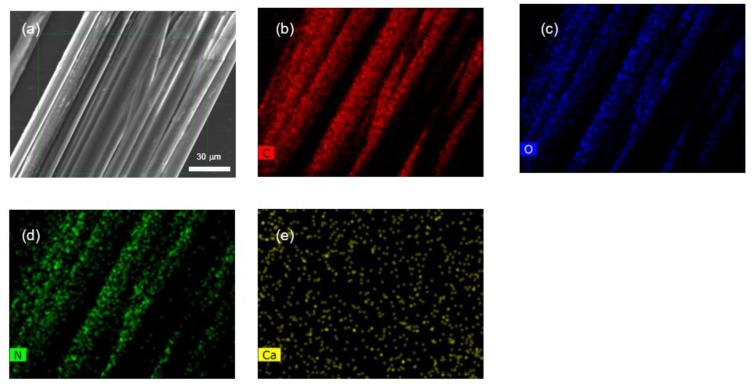
Elementary analysis of fibers produced by straining flow spinning. (**a**) Scanning electron micoscopy micrograph of regenerated silk fibers spun from the Ca-16% dope and its elementary energy dispersive X-ray spectroscopy (EDS) mapping for (**b**) carbon, (**c**) oxygen, (**d**) nitrogen, and (**e**) calcium. In spite of the high concentration of Ca^2+^ ions in the dope, no calcium remains in the fibers upon spinning to the resolution limit of the technique.

The high concentration of Ca^2+^ ions in the dope offers the possibility of checking the diffusion of different chemical moieties between the dope and the focusing fluid. In particular, the possible presence of calcium in the fibers can be assessed through EDS. Figure 4a shows a SEM micrograph of silk fibers spun from the 16%-Ca dope under the spinning conditions indicated above, and Figure 4b–e show the EDS mapping for the channels corresponding to carbon (Figure 4b), oxygen (Figure 4c), nitrogen (Figure 4d), and calcium (Figure 4e). As expected for protein fibers, the EDS mapping reveals the presence of carbon, oxygen, and nitrogen in the fibers, while no calcium is observed up to the resolution limit of the technique. This result shows that the Ca^2+^ ions initially present in the dope solution are removed as an effect of the interaction between the dope and the focusing fluid (possibly with the coagulant bath, too). The possible effect of this removal in the transition from protein solution to a solid fiber will be discussed in the next subsection.

### 5.2. Composition of the Focusing Fluid

Following the schematic depicted in Figure 2a, the interaction between the dope and the focusing fluid must create the conditions that favor the assembly of the proteins and eventually lead to the transition from the protein solution to the solid fiber. Since one of the key processes for the assembly of the proteins is the substitution of protein–water interactions for protein–protein interactions, the removal of water molecules from the dope is supposed to play a critical role in this transition. Water is removed from the dope by including a dehydrating agent in the composition of the focusing fluid, so that a competition is established between the fibroin proteins and the dehydrating agent for the water molecules initially present in the dope. A similar dehydrating mechanism is common in the conventional wet spinning of regenerated silk fibers and a number of alcohols are usually employed in the coagulating bath including methanol [29,30,31] and ethanol [56]. These alcohols differ in their affinity towards water molecules, which is higher in methanol, although at the expense of using a highly toxic chemical. In this regard, SFS allows avoiding the use of harsh or toxic solvents in the focusing fluid, so that the preferred choices are usually mild alcohols such as ethanol, isopropanol, and even, PEG.

However, the analysis of the natural spinning process shows that the formation of the solid fiber does not result exclusively from the mere removal of the water molecules that act as solvent in the dope. The presence of the pH switches at the N- and C-terminal domains of the protein imply that the protein conformation depends on the pH of the solution, as well as on the concentration of several ions. In turn, conformational changes do control the assembly process of the fibroins to a large extent. In this regard, and although the regenerated fibroins may suffer the cleavage of certain regions during degumming, the regenerated system is still sensitive to the physicochemical conditions of the protein solution.

Straining flow spinning offers the possibility of modifying the physicochemical conditions of the fibroins in a controlled way through the diffusion of chemical moieties from and to the dope and the focusing fluid. Thus, addition of a mild acid (acetic acid) to the focusing fluid appears to enhance the coagulating effect of the focusing fluid compared with the same composition with no added acid. Besides, as shown in Figure 4, the interaction between the dope jet and the focusing fluid promotes the removal of the Ca^2+^ ions, which stabilize the fibroin solution at concentrations higher than 8% (*w*/*v*), and is likely to contribute further to the formation of the solid fiber.

**Figure 5:**
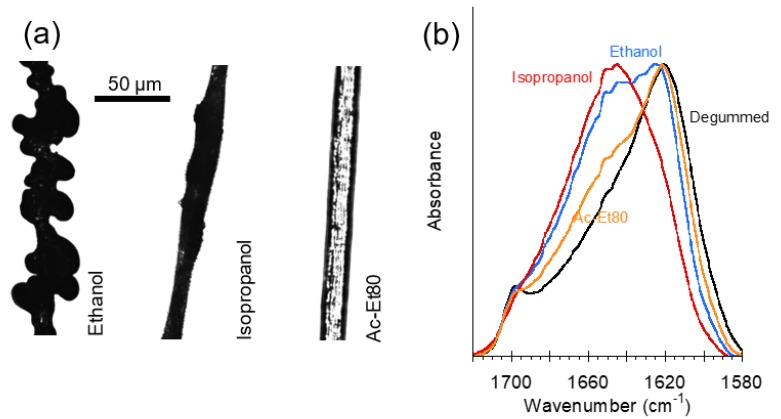
Morphology and Fourier-transform infrared spectroscopy (FTIR) spectra of regenerated silk fibers spun using different focusing fluid chemistries. (**a**) Morphology of the fibers observed using optical microscopy. (**b**) FTIR of the silk fibers in (**a**) shows amide I peaks between 1580 and 1720 cm^−1^. The FTIR spectrum of a degummed natural silkworm silk fiber (black line) is shown for comparison.

Figure 5a shows the effect of different compositions of the focusing fluid on the morphology and microstructure of the regenerated fibers. Three different compositions of the focusing fluid are compared: ethanol, isopropanol, and 80% ethanol/20% acetic acid solution in water (Ac-Et80). As shown in Figure 5, the homogeneity of the fibers largely depends on the focusing fluid used during the spinning process: fibers spun with ethanol appear as largely inhomogeneous, those spun with isopropanol appear slightly more homogeneous, and homogeneity is significantly improved when the Ac-Et80 mixture is used as focusing fluid. Usage of this latter focusing fluid implies a change in the pH from the initial value of pH ≈ 5.0 to a final value of pH ≈ 4.0.

The effect of different focusing fluids on the fibers is also apparent when the microstructure of the fibers is considered. Figure 5b compares the amide I peak obtained by Fourier-transform infrared spectroscopy (FTIR) of the regenerated fibers shown in Figure 5a and a degummed natural silkworm silk fiber. The amide I peak of a degummed natural silkworm silk fiber shows a peak at 1620 cm^−1^, which indicates the dominant contribution of the β-pleated secondary structure characteristic of silk fibers [25] to the amide I peak. In contrast, regenerated fibers spun with ethanol show a much broader peak due to the higher contribution of random coil and helical conformations at around 1640 cm^−1^ in comparison with the contributions of these secondary structure to the spectrum of the natural silk fibers. Interestingly, regenerated fibers spun with isopropanol exhibit a sharp peak, but displaced with respect to the natural fibers to a wavenumber of 1640 cm^−1^. Finally, a sharp peak at 1620 cm^−1^ is observed in regenerated fibers spun with Ac-Et80, although these regenerated samples present a shoulder at higher wavelengths that is not observed in the natural material suggesting a higher proportion of random coil and helical secondary structures in comparison with the natural material.

It is worth mentioning that the previous discussion on the requirements to the focusing fluid can be applied to a large extent to the coagulating bath fluid. As indicated above, very often the focusing fluid and the coagulating bath share a common composition, however, in contrast to other spinning techniques, SFS allows the employment of different chemistries for the focusing fluid and for the coagulating bath if necessary.

### 5.3. Influence of the Geometrical and Hydrodynamic Parameters

Besides the large flexibility for the production of regenerated silk fibers offered by SFS through the various combinations of dope and focusing fluid chemistries, the control exerted in the process through the hydrodynamic parameters is a feature characteristic of the technique. In addition, the hydrodynamic parameters can be varied in real time during the spinning process. This particularity of the technique was recognized in previous works where it was found that parameters such as the diameter of the fibers were largely controlled by the values of the flow rate of the dope and of the focusing fluid [47]. In this regard, the possibility of controlling the stresses exerted on the dope jet, which can be estimated from Equation (1), is a unique feature to SFS compared with other spinning techniques:

(1) $$\Delta P\approx \rho_{f}\frac{Q_{f}^{2}}{D_{1}^{4}}$$

where *ρ_f_* is the density of the focusing fluid, *Q_f_* is the flow rate of the focusing fluid, and *D*_1_ is the diameter of the nozzle outlet.

**Figure 6:**
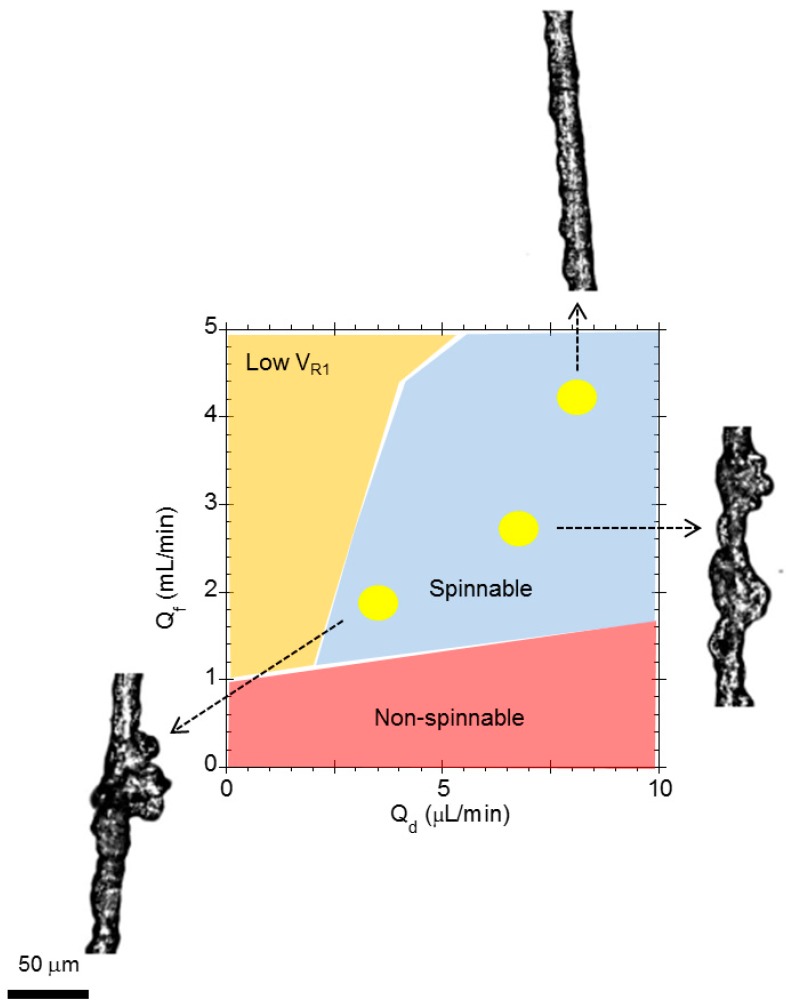
Chart of the spinning processes as determined by hydrodynamic parameters. The compositions of the dope and of the focusing fluid are kept fixed for all spinning processes in the chart. Three regions are defined and referred to as non-spinnable, spinnable, and low *V_R_*_1_. In the non-spinnable region, the fiber either does not form or breaks upon collection on the take-up roller. In the spinnable region, the fiber can be continuously spun and collected on the take-up roller. Finally, in the low *V_R_*_1_ region the fiber is formed, but accumulates in the coagulating bath. The morphologies of three fibers produced under different hydrodynamic conditions are shown, and the set of hydrodynamic conditions for spinning each fiber is indicated by a yellow circle in the chart. *Q_d_*: Flow rate of the dope; *Q_f_*: Flow rate of the focusing fluid; *V_R_*_1_: Speed of the take-up roller.

Figure 6 shows the influence of the hydrodynamic parameters: flow rate of the dope (*Q_d_*), flow rate of the focusing fluid (*Q_f_*), and velocity of the take-up roller (*V_R_*_1_) in a single spinning process. In all cases, a value of *V_R_*_1_ was fixed between 1 m/min and 4 m/min (the maximum *V_R_*_1_ of the take-up roller in the configuration of the spinning device) and the values of *Q_d_* and *Q_f_* were varied, while retrieving the fibers in the take-up roller. As shown in Figure 6, three regions can be defined: (i) at low values of *Q_f_*, there is a “non-spinnable” region, in which the fibers either do not form at all or break when transferred to the take-up roller; (ii) at high values of *Q_f_*, another region referred to as “low *V_R_*_1_” is defined, in which the rate of spinning is larger than the speed of the take-up roller and the fiber accumulates in the coagulating bath; and (iii) a “spinnable” region is defined, in which the fiber can be spun continuously and retrieved from the take-up roller. In addition to defining the conditions of the spinnable region, different values of the flow rate of the dope and of the focusing fluid may lead to fibers with different morphologies, as observed for the fibers spun under the conditions indicated by the yellow circles in Figure 6.

## 6. The Quest for High-Performance Regenerated Silk Fibers

Straining flow spinning offers a wide range of possibilities both in terms of the spinning conditions compatible with the technique and of the properties of the spun regenerated fibers. In this regard, the initial development of the technique followed two main guidelines: (i) the use of mild and environmentally friendly chemistries for both the dope and the focusing fluid (and/or coagulating bath); and (ii) the identification of a set of conditions that lead to the production of high-performance fibers with a work to fracture comparable to that of natural silkworm silk (≈70 MJ/m^3^). In particular, the conditions required to reach the high values of work to fracture (*W_f_*) were difficult to identify because of the enormous parameter space between the sets of spinning conditions summarized in Table 1. The selection of a suitable subset of processing parameters again followed the biomimetic approach used at the inception of SFS.

As indicated above, SFS allows spinning from regenerated silk fibroin with two different distributions of molecular weights, namely low and high molecular weights. Besides, fibers can be produced from dopes with protein concentrations as low as 4% (*w*/*v*), up to 16% (*w*/*v*), and possibly higher. Since the natural system requires extremely high molecular weight proteins at high concentrations, dopes were prepared from the high molecular weight regenerated fibroin at a concentration of 16% (*w*/*v*). Such a high concentration required the addition of 1 M CaCl_2_ to the dope solution to produce a stable protein solution in water.

Preliminary results from spinning processes using ethanol and isopropanol as focusing fluids showed that even these relatively mild dehydrating agents led to an abrupt solidification process, as revealed by the inhomogeneity observed in the fibers shown in Figure 5. Consequently, the usage of even milder coagulating chemistries was explored. In particular, a 30% PEG solution in water and a mixture of 80% ethanol and 20% acetic acid in water (Ac-Et80) with a final concentration of 0.2 M of acetic acid were employed both as focusing fluid and coagulating bath.

**Figure 7:**
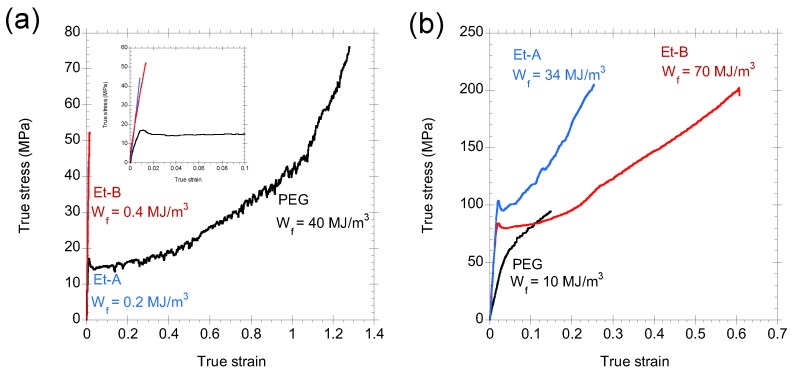
True stress–true strain curves of regenerated silk fibers produced by straining flow spinning and tested in air. Coagulating bath and focusing fluid for Et-A and Et-B samples: mixture of 80% ethanol and 20% acetic acid solution. Coagulating bath and focusing fluid for polyethyleneglycol (PEG) sample: 30% PEG in water. (**a**) As-spun fibers. The inset shows the true stress–true strain curves at low values of strain. (**b**) Wet-stretched fibers after drying. The spinning conditions are indicated in the text and the work to fracture (*W_f_*) of each fiber is shown here.

Finally, after fixing the chemistries of the dope and of the focusing fluid, a screening was performed on the geometrical and hydrodynamic parameters of the system [47] in order to identify the set conditions that led to high-performance fibers. Three sets of spinning conditions were selected and combined either with PEG [45] (Figure 7) or with the Ac-Et80 mixture [45,48] (referred to as Et-A and Et-B, respectively; Figure 7). In this case, Et-B fibers were produced with a flow rate of the focusing fluid (*Q_f_* = 2.5 mL/min) that is five times higher than that of the Et-A condition (*Q_f_* = 0.4 mL/min). 

Figure 7a compares the true stress–true strain curves of the as-spun fibers using the three sets of conditions. The insert shows a close-up of the curves at low true strain values. As observed with the morphology, the fracture surfaces and microstructure of the fibers, large differences in the tensile properties of the fibers are observed as a function of the detailed processing parameters. In particular, the PEG sample shows a remarkable value of work to fracture of *W_f_* = 40 MJ/m^3^ (half of the value of the natural material) that, to the best of our knowledge, is the highest work to fracture of any regenerated silkworm silk fiber not subjected to any post-spinning treatment. In contrast to this high value, the samples coagulated in Ac-Et80 show a brittle behavior until fracture with values of *W_f_* smaller than 1 MJ/m^3^.

Previous works on regenerated silkworm silk fibers showed that the tensile properties of initially brittle fibers can be considerably improved by subjecting the fibers to a wet-stretching process (see Section 2). Consequently, fibers spun with the three sets of conditions were wet-stretched up to a 90% of their breaking strain in water, allowed to dry, and tested. The true stress–true strain curves of the wet-stretched fibers are shown in Figure 7b. It is apparent that wet-stretching worsens the tensile properties of the PEG sample, but it increases the mechanical behavior of the Et samples (i.e., samples with Ac-Et80 focusing fluid and coagulant) considerably. Thus, sample Et-A reaches a remarkable value of *W_f_* = 34 MJ/m^3^ and sample Et-B reaches a value of *W_f_* = 70 MJ/m^3^, comparable to that found in the natural material. Wet-stretching is an efficient way of modifying the tensile properties of the fibers in the laboratory, but it is difficult to implement on an industrial scale. Consequently, subsequent studies were undertaken that proved the equivalence between the wet-stretching process and a post-spinning drawing step during the processing of the fibers [48]. Therefore, the combination of the basic SFS setup and a post-spinning drawing step constitutes an efficient procedure for the mass production of high-performance regenerated silk fibers.

**Figure 8:**
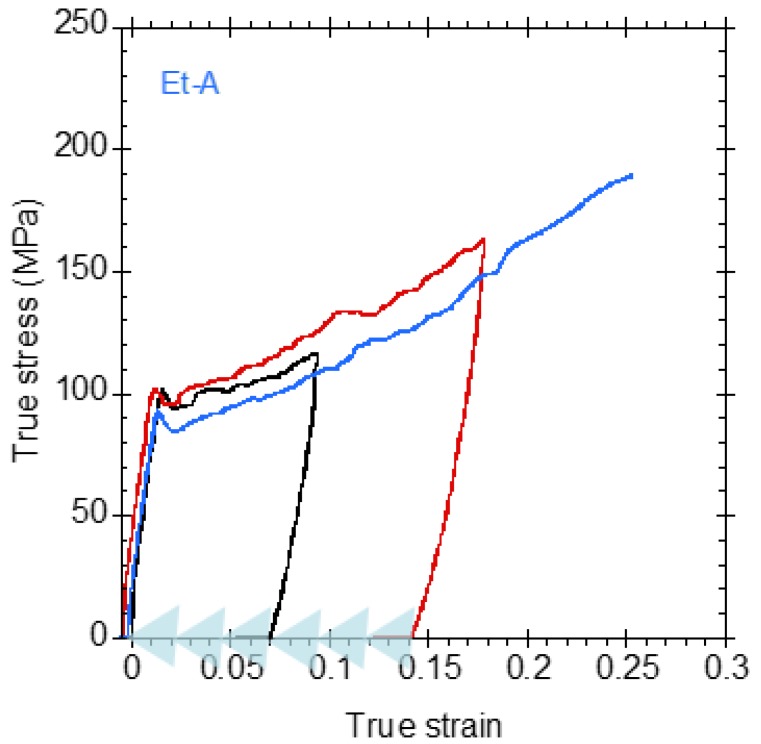
Recovery test of an Et-A regenerated silk fiber. Coagulating bath and focusing fluid for Et-A: mixture of 80% ethanol and 20% acetic acid solution. The fiber is stretched in air in a first cycle up to a strain of 0.1 (black curve), immersed in water and allowed to supercontract. Subsequently, the fiber is stretched up to a strain of 0.18 (red curve) and allowed to supercontract again. The contraction steps in water between each cycle of stretching in air are indicated by the light blue arrows on the true strain axis. Finally, the fiber is tensile tested up to breaking (blue curve). The concurrence of all the curves proves the supercontraction ability of this fiber.

Finally, several previous studies had shown that regenerated silkworm silk fibers can be endowed with the ability to supercontract [35], a feature characteristic of spider silk, which is not exhibited by natural silkworm silk fibers. Supercontraction implies the existence of a ground state, to which the fiber can return independently from its previous loading history by being immersed in water. Supercontraction is assessed through recovery tests in which the fiber is stretched to a certain value of strain, and then is allowed to contract in water, dried, and tested in air. The concurrence of the true stress–true strain curves after the fiber is allowed to supercontract and dried is the evidence that the fiber shows a proper ground state. Figure 8 shows a recovery test on a fiber spun under Et-A conditions and subjected to two stretching supercontraction cycles, before being tested to breaking. The immersion in water and contraction step after being stretched in air is indicated by the light blue arrows in Figure 8. As indicated above, the concurrence of the true stress–true strain curves is an indication of the existence of a ground state in the Et-A regenerated silkworm silk fibers.

## 7. Conclusions

Straining flow spinning represents an efficient biomimetic approach for the production of silk-based artificial fibers. The formation of the solid fiber from the protein solution is the result of the interaction of the dope jet with a second fluid (focusing fluid), and possibly, with a coagulating bath. In turn, the interaction between both fluids is controlled by the geometrical setup of the device, as well as by the hydrodynamic parameters employed. The large number of processing parameters imparts SFS with a versatility superior to most other silk spinning techniques.

This versatility, in turn, was exploited to guide the development of the technique by two basic principles: (i) Fibers had to be produced under environmentally friendly conditions, both in terms of the composition of the dope and focusing fluid and on the physicochemical parameters (i.e., pH or concentration of Ca^2+^ ions) required by the process; and (ii) it was necessary to find a set of processing conditions that led to the production of high-performance fibers. This review shows how both principles were fulfilled, so that high-performance silk fibers can be produced efficiently from silkworm silk fibroin solutions through SFS with the exclusive use of mild chemistries.

Although our previous studies have shown the possibilities of this technique, they have concentrated on a very limited range within the wide variety of possible conditions and opportunities. Thus, there are complete areas that remain unexplored, such as spinning from solutions of either natural spider silk spidroin or recombinant bioinspired silk proteins, just to mention a couple exciting possibilities. Consequently, a deeper exploration of SFS will probably have a significant influence in the future development of biomimetic production systems of high-performance and biocompatible fibers. These new materials, in turn, should have a significant impact on the production of new meta-materials, fabrics, scaffolds, and a large variety of products for numerous applications in the future. 

## 8. Patents

Patent application of the SFS technology [57]. The SFS technology is licensed to Silk Biomed, S.L.

## Figures and Tables

**Table 1:** Summary of the geometrical, hydrodynamic, and chemical parameters that define a straining flow spinning process.

Geometrical	Hydrodynamic	Chemical
Capillary inner and outer diameter Nozzle outlet diameter Capillary–nozzle outlet distance Tapering angle of the capillary Convergent profile of the nozzle outlet region	Flow rate of the dope Flow rate of the focusing fluid Velocity of the take-up roller Velocity of post-spinning roller	Composition of the dope Composition of the focusing fluid Composition of the coagulating bath
